# Temporal, Plant Part, and Interpopulation Variability of Secondary Metabolites and Antioxidant Activity of *Inula helenium* L.

**DOI:** 10.3390/plants8060179

**Published:** 2019-06-17

**Authors:** Nenad Zlatić, Dragana Jakovljević, Milan Stanković

**Affiliations:** Department of Biology and Ecology, Faculty of Science, University of Kragujevac, 34000 Kragujevac, Serbia; nzlatic@gmx.com (N.Z.); mstankovic@kg.ac.rs (M.S.)

**Keywords:** practical importance, antioxidant activity, phenolic compounds, quantitative analysis, spatio-temporal variability, environmental factors

## Abstract

Variations in abiotic environmental factors have significant effects on quantity and quality of secondary metabolites, which is particularly important for plant species that possess biologically active compounds. The purpose of this study is determination of the total phenolic content, flavonoid concentration, and antioxidant activity of the different parts of *Inula helenium* L. (Asteraceae) sampled from different populations and in different time periods. The amounts obtained for the total phenolics varied from 16.73 to 89.85 mg of gallic acid (GA)/g. The concentration of flavonoids ranged from 9.32 to 376.22 mg of rutin (Ru)/g. The IC_50_ values of antioxidant activity determined using the 1,1-diphenyl-2-picrylhydrazyl (DPPH) free radical method varied from 161.60 to 1563.02 μg/ml. The inflorescence and roots possessed high concentration of phenolic compounds and significant antioxidant activity, while leaves contained the highest concentration of flavonoids. Additionally, the quantity of the phenolics, as well as antioxidant activity, significantly varied among the different populations due to different impacts of environmental factors. This research showed that *I. helenium* represents an abundant source of bioactive substances, and that the quantity of these compounds greatly differs among the different populations as well as in the same populations regarding the different time periods as well as plant parts.

## 1. Introduction

As a part of adaptive strategies to the variability of environmental factors, plant secondary metabolites have major roles in the adaptation of plants to unfavorable conditions [1]. Secondary metabolites, therefore, participate in the process of the adaptation of plants to environmental conditions, and their quantity varies depending on the numerous ecological factors to which the plant is exposed [2,3], including temperature, humidity, light intensity, water supply, and minerals [4]. Phenolic compounds represent one of the three main groups of plant secondary metabolites [5]. Among these compounds, phenolics are particularly important due to their ability to eliminate free radicals and to function as antioxidants. Likewise, secondary metabolites are attributed with numerous protective functions in the human body, including antioxidative, antibiotic, anticarcinogenic, and pharmacological effects [6]. Health promoting effects of plant-derived secondary metabolites have been well documented [6,7,8,9].

The genus *Inula* L. (Asteraceae) includes species native to Europe, Africa, and Asia, and naturalized in America, among which *Inula helenium*, *I. salicina*, and *I. britannica* are best-known. Elecampane (*I. helenium* L.) is a perennial herbaceous plant in which oval and elongated leaves without petioles grow on moderately branched shoots. *I. helenium* primarily populates warm, damp, open, and calcareous habitats near river and stream banks in beech and conifer forests; however, due to wider application, the successful growth of *I. helenium* has been enabled on territories outside its initial area [10]. Traditionally, *I. helenium* is used in the treatment of arthritis, diabetes, rheumatism, pulmonary tuberculosis, and acute respiratory diseases [11]. Apart from the given therapeutic effects, detailed investigations in recent years showed that *I. helenium* possesses significant amounts of bioactive compounds such as inulin, sesquiterpene lactones [12,13], phenolic acids, flavonoids, and terpenoids [14] with antibacterial [7], antiparasitic, antioxidant [15,16], and anticancer activities [8].

The determination of spatial and temporal dynamics in the synthesis of secondary metabolites, together with their quantitative variability and activity, has scientific and practical significance and provides an increase of exploitation of plant material from their natural habitats. On this basis, the elementary and primary goal of the research is the evaluation of time-conditioned, interpopulation, and variability among the plant organs by applying analysis of the total concentration of phenolics, flavonoids, and antioxidant activity of the *I. helenium* extracts.

## 2. Results

The investigations of content of secondary metabolites and antioxidant activity of *I. helenium* was conducted using different plant parts (root, stem, leaves, and inflorescences) from two localities (named Locality 1 and Locality 2) and in different time periods from Locality 1 (1a and 1b).

### 2.1. Concentration of Total Phenolic Compounds

The total concentration of phenolic compounds obtained in the ethanol extracts of the plant organs of the *I. helenium* is illustrated in Table 1.

The total concentration of phenolic compounds in the plant extracts obtained from the organs of *I. helenium* varied from 16.73 to 89.85 mg of gallic acid (GA)/g. Regarding the plant parts, the greatest quantity of phenolics was measured in the extracts obtained from inflorescences (89.85 mg of GA/g), whereas the concentration of phenolic compounds in roots and leaves were similar. The lowest quantity of phenolics was observed in the stem extracts. While analyzing the values obtained from the samples collected from the same locality but in different time periods, it was detected that the values of the total concentration of phenolic compounds slightly decreased. Additionally, the values of the samples taken from Locality 2 were the lowest.

### 2.2. Concentration of Flavonoids

The flavonoid concentration of *I. helenium* extracts is shown in Table 2.

The quantity ranges between 9.32 and 376.22 mg of rutin (Ru)/g of the extracts with the highest flavonoid concentration measured in the ethanol extract of leaves, whereas the values of flavonoids were lower in the roots. While observing the values of the samples taken from the same locality but in different time period, it was noticed that the values of the total concentration of flavonoids greatly decreased. In addition, the values obtained from the second locality followed the trend of low values. In both cases, the same trend was observed, since the highest concentration of flavonoids was observed in the leaf extracts and the lowest in extracts obtained from roots.

### 2.3. Antioxidant Activity

The results of antioxidant activity of plant parts extracts from *I. helenium* are illustrated in Table 3.

The antioxidant activity of the plant organs of the *I. helenium* varied from 161.60 to 1563.02 μg/ml of the extract. Among the different plant parts, root samples demonstrated the largest capacity to neutralize DPPH radicals. Significant antioxidant activity was also noticed in the case of the inflorescence extract from Locality 1a (IC_50_ = 183.95 μg/ml). The stem extracts showed the lowest capacity to neutralize free radicals. In addition to the plant parts, sampling time, together with locality, influenced the capacity of *I. helenium* extracts to neutralize free radicals, since the highest antioxidant activity was measured in extracts from roots sampled from Locality 1a, while antioxidant capacity decreased in samples from Locality 1b and Locality 2.

### 2.4. Correlation Coefficient

The results of the degree of correlation between plant parts, localities, and type of analysis are presented in Table 4.

Based on the results presented in Table 4, it was shown that there was a significant correlation between the investigated parameters. The values of correlation between the examined compounds and the antioxidant activity for localities showed that there was a significant correlation between the quantity of phenolic compounds and antioxidant activity for all localities, while no significant correlation was shown between total flavonoids and antioxidant activity.

## 3. Discussion

Phenolic compounds are highly soluble in polar solvents [17] and among them, ethanol is one of the most effective solvents for polyphenolic compounds and it is safe for human use [18,19].

The primary role of plant secondary metabolism is adaptation to different ecological factors, and products of secondary metabolism are conditioned by plant primary metabolism [20]. Locality 1 is a mesophilous habitat, i.e., a meadow located at an altitude of 973 m. Locality 2 is a hygrophilous habitat, i.e., the edge of the forest in the vicinity of the stream located at an altitude of 447 m. The analysis of the content of secondary metabolites among the sampled populations growing at different altitudes showed significant difference between populations in the quantity of the investigated metabolites. The plant species in the habitats with the increased intensity of light contain higher content of phenolics since these metabolites have considerable influence on the adaptational abilities of plants populating these habitats and, therefore, the quantity of phenolics in a certain species increases proportionally with the higher altitudes. The increased quantity of phenolic compounds in plants plays a protective role against UV-b rays, which are more intense at higher altitudes [21,22]. Numerous studies have confirmed that plants produce larger quantities of secondary metabolites in order to protect protein photosystem II and DNA from harmful UV radiation [23]. It has been demonstrated that the more intense exposure of the species *Catharanthus roseus* to UV-b radiation brings about greater production of certain secondary metabolites, such as vincristine and vinblastine [24].

It was noticed that there were certain differences in the total concentration of phenolic compounds in the plant parts of *I. helenium* with the highest concentrations of phenolics observed in the flower extract (89.85 mg of GA/g). Similar results were obtained by analyzing the species *Euphorbia helioscopia* L., where the highest concentration of phenolic compounds was in the flower extract [25]. The authors who performed the comprehensive analysis of qualitative–quantitative composition of plant organs showed the difference in values of these compounds in flowers, leaves, stem, and root. The concentration of phenolic compounds in a particular plant organ depends upon the phenolic synthesis in those organs. The variation in the quantity of these compounds relies on the difference in morphological and anatomical structures, as well as in the numerous physiological processes that occur in different plant organs [26].

Further observations of our study revealed the difference in the total concentration of the phenolic compounds based on the time of the sampling. The samples from the Locality 1, sampled in July, had greater quantities of phenolics in comparison with samples from the same locality but sampled in October. The climatic differences in the given period may speed up or slow down the process of accumulation of phenolics in plants and their organs. The difference in seasonal variability of the concentration of phenolic compounds of a certain species depends upon the length of day and the differences in day and night temperatures [27]. Phenological changes are mostly influenced by differences between spring and summer temperatures [28]. This fact is highly relevant in terms of plant metabolism and synthesis of phenolic compounds.

In addition to total phenolics, the results also revealed the difference in the concentration of flavonoids since the quantity of flavonoids increased with an increase in altitude. As has been shown, the influence of UV-b rays increases the production of flavonoids in barley [29].

The leaves of the *I. helenium* contained the greatest amounts of flavonoids (376.22 mg of Ru/g). Flavonoids belong to the largest group of phenolic compounds and can be found in all plant parts, particularly in the cells of the photosynthetic apparatus and accordingly, in leaves [9]. The fact that the leaf extracts contained the highest concentration of flavonoids is in accordance with numerous studies [30,31]. The research demonstrated that the species that grew in a mesophilous habitat produced a greater quantity of flavonoids in comparison with the species in a hygrophilous habitat. Likewise, *Ranunculus acris* growing in semi dry habitats had greater leaf area than those in a wet habitat [32].

The quantity of flavonoid variation is greatly influenced by the time of sampling of *I. helenium*. The species collected in Locality 1 in July contained higher concentrations of flavonoids in comparison with the samples taken in October. Previous studies on *Salvia fruticosa* Mill. and *Rosmarinus officinalis* L. demonstrated that the difference in the quantity of flavonoids greatly depends upon the group of ecological factors and the impact they have in a certain habitat [33].

The results for antioxidant activity of the extracts from the *I. helenium* established the differences between the populations sampled at different altitudes. The plant population sampled from Locality 1 (at an altitude of 973 m) showed greater antioxidant activity in all plant parts in comparison to the plant population sampled from Locality 2 (at an altitude of 447 m). These results are in accordance with the studies that pointed out that the samples collected at higher altitudes had better antioxidant activity than the extracts of the samples taken at lower altitudes [22,34].

The highest value of antioxidant activity was observed in the root extract (161.60 μg/ml). Previous investigations demonstrated that the main phenolic compounds in the root extract are phenolic acids (caffeic, chlorogenic, dicaffeoyl quinic, hydroxibenzoic); terpenes (alantolactone); and different flavonoids (epicatechin, catechin gallate, ferulic acid-4-O-glucoside, dihydroquercetin pentosyl rutinoside, kaempherol-7-O-dipentoside, quercetin-3-O-β-glucopyranoside) [16,35], among which the root extract of the *I. helenium* contains the most substantial quantity of caffeic acid [16]. Bearing in mind that these phenolic acids show high antioxidant values due to the interruption of the reaction of oxidation, the DPPH scavenging capacity of extracts may be predominantly due to their phenolic hydroxyl groups.

The relations among the content of secondary metabolites and antioxidant activity have already been established for several plant species [36,37]. To further instantiate the correlation, plant parts of different *Teucrium* species [38,39] showed significant linear correlation between the concentration of phenols and antioxidant activity. The correlation between the total phenolic content and antioxidant activity in the extracts of plant parts have negative value since the increase in the quantity of the analyzed compounds was accompanied by the decrease of the antioxidant activity. In other words, the lower the IC_50_ values are, the greater the antioxidant efficiency is. The obtained values for the correlation between the quantity and the antioxidant activity of phenolic compounds suggest that the phenolic compounds are the principal active substances and the bearers of the antioxidant activity. Therefore, the phenolic content of plants may contribute directly to the antioxidant activity of extracts [37].

Seasonal variations in the amount of flavonoids are demonstrated by Alías et al. [40]. According to these authors, the concentration of flavonoids is conditioned by climate characteristics (particularly temperature), which indicates the ecological functions of these metabolites. The time–space variability in the production of secondary metabolites could affect the ecological interactions of the species and their ecophysiological behavior, which varies depending on the season and the plant organ [41]. Interpopulation variations have advantage since the species will be more likely to survive changes with the adequate amount of compounds to respond to adverse environments [42].

## 4. Materials and Methods

### 4.1. Plant Material

The plant material was sampled from different localities in Western Serbia (Table 5). Locality 1a was the same locality as Locality 1b with the only difference being the samples were taken in different time periods. The localities from which the species were sampled were 40 km apart from each other, and these localities differed in the type of habitat and the group of ecological factors predominant in the habitats. Under these environmental conditions, the flowering period of *I. helenium* is from the beginning of June to the end of October. The voucher specimen of *I. helenium* was deposited in the Herbarium at the Department of Biology and Ecology, Faculty of Science, University of Kragujevac. Collection of plant material was carried out by sampling flowering branches from several representative individuals of the population. The plant material was air-dried in a dark place and at ambient temperature. The dried material was powdered and stored in sealed containers until use.

### 4.2. Preparation of Plant Extracts

One gram was measured from every sample of the ground plant material. The plant material was transferred to an Erlenmeyer flask into which 30 mL of ethanol was added. Previous research established ethanol as a prominent solvent of the extraction of bioactive compounds from *I. helenium* [43,44]. The extraction was performed using a Soxhlet apparatus. The obtained extract was preserved in a cold and dark place.

### 4.3. Determination of Total Phenolics in the Plant Extracts

The total phenolic concentration was determined using gallic acid as a standard [45]. The reaction mixture (0.5 mL of the plant extract, 2 mL of 7.5% NaHCO_3_, and 2.5 mL of 10% Folin–Ciocalteu reagent) was incubated at 45 °C for 45 min and the absorbance was measured at 765 nm. The total phenolic content was expressed in terms of gallic acid equivalent (mg of GA/g of extract).

### 4.4. Determination of Total Flavonoids in the Plant Extracts

The concentration of flavonoids was determined using rutin as a standard [46]. The test sample contained 1 ml of the extract solution and 1 ml of 2% AlCl_3_. The samples were incubated at 20 °C for 60 min and the absorbance was measured at 415 nm. The concentration of flavonoids was expressed in terms of rutin equivalent (mg of Ru/g of extract).

### 4.5. Evaluation of Antioxidant Activity

The efficiency of the plant extract to scavenge 1,1-diphenyl-2-picrylhydrazyl (DPPH) free radicals was evaluated using the spectrophotometric method [47], accepted with adequate modifications [39]. The IC_50_ values were estimated through the non-linear regression analysis using the % inhibition versus the concentration sigmoidal curve. In presented results, antioxidant efficiency of the extracts increased with the decreasing of IC_50_ values.

### 4.6. Statistical Analysis

Statistical analysis was performed using the SPSS (Chicago, IL) statistical software package (SPSS for Windows, version XII, 2008). Data are expressed as arithmetic mean of three analyses ± standard deviation. Comparisons were done using the Mann–Whitney U test (*p* < 0.05 or 0.01). Correlation coefficient (r) was used to study the relationship between variables.

## 5. Conclusions

The results of this research demonstrate that there is a significant variation in the quantity of secondary metabolites of *I. helenium*, which was sampled in different populations, among different plant organs, and in different time periods. The research provides evidence that the plant organs of the *I. helenium* are rich in phenolic compounds and that the greatest concentration of phenolic compounds was found in inflorescences. The highest concentration of flavonoids was observed in leaves, whereas the most expressive antioxidant activity was detected in the root and inflorescence. In addition, the concentrations of secondary metabolites in *I. helenium* depend on the time period of sampling. The increased quantity and activity of phenolic compounds in the samples of plant populations from the high-altitude locality prove the claim that phenolics are vital in terms of ecophysiological adaptation of the species *I. helenium* to the specific ecological conditions predominant in the habitats from which the samples were collected. Obtained results show a significant role for the exploitation of plant material sampled from natural or cultivated habitats.

## Figures and Tables

**Table 1:** Total phenolic content in the different parts of *I. helenium* (mg of gallic acid (GA)/g).

Locality	Root	Stem	Leaves	Inflorescence	*p*
Locality 1a	71.24 ± 0.49	27.43 ± 0.12	69.02 ± 0.51	89.85 ± 1.38	a
Locality 1b	67.22 ± 0.80	26.35 ± 0.25	67.08 ± 0.69	88.60 ± 1.27	a
Locality 2	44.40 ± 1.18	16.73 ± 0.18	49.61 ± 1.04	61.06 ± 0.57	b *

Each value is the average of three analyses ± standard deviation. *p* is the significance obtained by the Mann–Whitney U test; different letters indicate significant differences between localities; the asterisk is for significant level *p* < 0.01.

**Table 2:** Flavonoid content in the different parts of *I. helenium* (mg of rutin (Ru)/g).

Locality	Root	Stem	Leaves	Inflorescence	*p*
Locality 1a	20.27 ± 0.29	66.88 ± 0.39	376.22 ± 2.88	41.47 ± 1.61	a
Locality 1b	12.56 ± 0.19	29.01 ± 0.37	250.86 ± 3.35	38.23 ± 0.33	ab
Locality 2	9.32 ± 0.21	20.04 ± 0.61	191.20 ± 1.25	25.42 ± 0.56	b

Each value is the average of three analyses ± standard deviation. *p* is the significance obtained by the Mann–Whitney U test; different letters indicate significant differences between localities (*p* < 0.05).

**Table 3:** Antioxidant activity of the different parts of *I. helenium* (IC_50_, µg/ml).

Locality	Root	Stem	Leaves	Inflorescence	*p*
Locality 1a	161.60 ± 2.11	619.73 ± 3.05	338.83 ± 2.95	183.95 ± 1.51	a
Locality 1b	198.01 ± 1.84	842.05 ± 4.16	573.82 ± 3.12	222.25 ± 1.92	a
Locality 2	285.10 ± 2.35	1563.02 ± 6.77	865.32 ± 4.88	427.35 ± 2.08	b *

Each value is the average of three analyses ± standard deviation. *p* is the significance obtained by the Mann–Whitney U test; different letters indicate significant differences between localities; the asterisk is for significant level *p* < 0.01.

**Table 4:** Correlation coefficient (r) between the quantity of TP (total phenolic compounds), TF (total flavonoids), and AA (antioxidant activity) comparing plant parts and localities.

**Plant Parts**				
		TP	TF	AA
Root	TP	1	0.816	−0.988
	TF	-	1	−0.894
	AA	-	-	1
Stem	TP	1	0.715	−0.990
	TF	-	1	−0.803
	AA	-	-	1
Leaves	TP	1	0.805	−0.932
	TF	-	1	−0.965
	AA	-	-	1
Inflorescence	TP	1	0.988	−0.994
	TF	-	1	−0.998
	AA	-		1
**Localities**				
		TP	TF	AA
Locality 1a	TP	1	0.035	−0.931
	TF	-	1	0.143
	AA	-	-	1
Locality 1b	TP	1	0.138	−0.863
	TF	-	1	0.265
	AA	-	-	1
Locality 2	TP	1	0.249	−0.834
	TF	-	1	0.114
	AA	-	-	1

**Table 5:** General sampling data of *I. helenium*.

Sample	Locality	Type of Habitat/Vegetation	Altitude and Exposure	Latitude and Longitude	Date of Sampling
Sample 1	Locality 1a (Bratljevo)	Mesophilous habitat, the edge of the forest	973 m, W	43°28′32″ N 20°10′24″ S	12 July 2014
Sample 2	Locality 1b (Bratljevo)	Mesophilous habitat, the edge of the forest	973 m, W	43°28′32″ N 20°10′24″ S	19 October 2014
Sample 3	Locality 2 (Prilike)	Hygrophilous habitat, forest stream bank	447 m, W	43°37’50" N 20°8′44″ S	12 July 2014
